# Quantifying Temperature and Osmotic Stress Impact on Seed Germination Rate and Seedling Growth of *Eruca sativa* Mill. via Hydrothermal Time Model

**DOI:** 10.3390/life12030400

**Published:** 2022-03-09

**Authors:** Sheharyar Khan, Abd Ullah, Sami Ullah, Muhammad Hamzah Saleem, Mohammad K. Okla, Abdulrahman Al-Hashimi, Yinglong Chen, Shafaqat Ali

**Affiliations:** 1 Department of Botany, University of Peshawar, Peshawar 25120, Pakistan; sheharyarbotany@uop.edu.pk; 2 Xinjiang Key Laboratory of Desert Plant Roots Ecology and Vegetation Restoration, Xinjiang Institute of Ecology and Geography, Chinese Academy of Sciences, Urumqi 830011, China; abdullahbotany123@gmail.com; 3 State Key Laboratory of Desert and Oasis Ecology, Xinjiang Institute of Ecology and Geography, Chinese Academy of Sciences, Urumqi 830011, China; 4 Cele National Station of Observation and Research for Desert-Grassland Ecosystems, Cele 848300, China; 5 College of Plant Science and Technology, Huazhong Agricultural University, Wuhan 430070, China; saleemhamza312@webmail.hzau.edu.cn; 6 Department of Botany and Microbiology, College of Science, King Saud University, Riyadh 11451, Saudi Arabia; malokla@ksu.edu.sa (M.K.O.); aalhashimi@ksu.edu.sa (A.A.-H.); 7 The UWA Institute of Agriculture, UWA School of Agriculture and Environment, The University of Western Australia, Perth, WA 6001, Australia; yinglong.chen@uwa.edu.au; 8 Department of Environmental Sciences and Engineering, Government College University, Allama Iqbal Road, Faisalabad 38000, Pakistan; 9 Department of Biological Sciences and Technology, China Medical University, Taichung City 40402, Taiwan

**Keywords:** *Eruca sativa*, germination, cardinal temperature, hydrothermal constant, water stress

## Abstract

Germination models are quite helpful in predicting emergence times, dormancy periods, and their applications in crop management. This study investigated the germination behaviors of *Eruca sativa* Mill. in response to fluctuations in temperatures (*T_s_*) and water potentials (*ψ_s_*). Germination percentage (GP) increased 95% with rising temperature within the range of 20–30 °C, and decreased 25% at 5 °C. Moreover, each *ψ* and *T* resulted in a decrease in GP as *ψ* decreased. Further, we noted that the *θT*1 value was substantially high at 30 °C and in (0 MPa), whereas the *θT*2 value was maximum at 10 °C (−0.02 MPa) and it decreased with decreasing *Ψ*. The maximum hydrothermal time constant (*θ*HTT) and hydrotime (*θ*H) values were obtained at 10 and 30 °C, respectively. In addition, a linear increase in the GR_(*g*)_ pattern was observed at *T_b_* and a decrease below the *T_o_*. The calculated cardinal *T_s_* was 5 °C for the base *T*, and 30 °C for both the optimum and ceiling *T*. The germination characteristics were higher at 30 °C having (0 MPa). Therefore, using cardinal temperatures, germination results, and the hydrothermal time model (HTT) could reveal the independent and interactive impacts of both *T* and the *Ψ* on the response of seed germination subjected to diverse environmental conditions.

## 1. Introduction

The plant *Eruca sativa* Mill. (family: Brassicaceae) is a herbaceous industrial crop plant cultivated worldwide. It is primarily consumed fresh for its characteristic spicy flavor. Moreover, it is rich in health-promoting compounds with numerous medicinal properties [1]. The different extracts of this plant have been found to have anti-cancer, antioxidant, antithrombotic, and anti-inflammatory properties [2,3,4,5,6]. *E. sativa* contains a high level of erucic acid, making it a potential source of industrial oils [7]. The continuous change in climatic conditions has led to an increase in the frequency and intensity of abiotic stress factors that negatively impact plant growth, development, and yields [8,9,10,11].

The germination of seeds is a complex physiological phenomenon in the development of plants that is susceptible to abiotic factors including temperature, salinity, water potential, drought, and flooding [12,13,14,15]. Modeling seedling growth develops a quantitative description of how and why seeds germinate differently under various environmental conditions. For example, the effects of both temperature (*T*) and water potential (*ψ*), independently or interactively (*T* × *ψ*), impact germinating seeds studied with hydrotime, thermal time, and hydrothermal time models [16]. *T* and *ψ* are two key environmental stress factors that affect germination rate, germination percentage, and seedling emergence [17,18]. A hydrotime model estimates how seed germination is affected by water potential [19,20].

For instance, the germination process may be slowed or halted due to changes in the water potential of the permeation medium [21,22]. SG and sapling mortality are frequently caused by inadequate soil moisture, which initiates seed embryo metabolism and development [23]. The germination rate (GR) has a direct correlation with the variability between the surrounding *ψ* and the physiological threshold *ψ* for the emergence and growth of seedlings [16], and species distribution [24]. When there is no water limitation in the field, soil temperature also plays a significant role in seed germination and subsequent plant establishment [25]. When identifying the appropriate planting date for each crop, it is critical to know the three cardinal *T_s_* of SG, namely *T_b_* (low temperature; at which SG is equal to zero); *T_o_* (optimum temperature; at which SG is maximum); and *T_c_* (maximum or ceiling temperature; at which SG is equal to zero) [23,26,27]. Due to extreme temperature fluctuations and low atmospheric humidity and rainfall, water shortages are becoming a major concern for plant growth and productivity [28]. In order to optimally manage crop plants, including *E. sativa*, in changing climate conditions, it is important to gain a deeper understanding of several basic aspects, such as its geographical distribution, seed germination behavior, and seedlings’ metabolism and growth.

In terms of forecasting the behavior of seed germination and seedlings’ emergence to abiotic stress, the use of different models can be quite useful. For instance, the hydrotime (HT) and hydrothermal time models (HTT) are used to examine the response of seed germination to fluctuating *ψ* and *T* independently or interactively (*ψ* × *T*) [29]. The HTT model is a population-based threshold model that can characterize the concept of germination time to fluctuating *ψ* and *T* in the sub-optimal range (from *T_b_* to *T_o_*) and supra-optimal range (from *T_o_* to *T_c_*) [30]. Several plants, namely *Sinapis arvensis* L., *Plantago ovata* Forssk., Safflower, *Papaver somniferum* L., and *Lathyrus* spp., have adopted this approach so far [20,31,32].

This study aimed to investigate whether TT, HT, and HTT models can be used simultaneously to identify rocket seed germination responses to fluctuating *ψ* and *T*, independently or interactively (*ψ* × *T*). Evaluating seed behavior and seedling emergence will provide us with valuable insight into *E. sativa* productivity under continuously changing climatic conditions and/or its distribution around the world.

## 2. Materials and Methods

### 2.1. Seed Sowing and Stress Implementations

The rocket seeds (*Eruca sativa* Mill.) were generously contributed by the Pakistan Forest Institute (PFI), Khyber Pakhtunkhwa (KP) Pakistan. After being treated with 95% ethanol (3 min), the seeds were washed with double distilled water and dried at a normal temperature under shade conditions. A Petri dish experiment was performed following the randomized complete block design (RCBD), at the Department of Botany, University of Peshawar, KP, Pakistan, by subjecting seeds to fluctuating *Ψs* (0, −0.01, −0.02, and −0.05 MPa) [33] and *T* (5, 10, 15, 20 and 30 °C). There were 40 seeds per Petri dish on the Wathman No. 1 filter paper, dampened with double distilled water (5 mL) and PEG6000 solutions. Each treatment was repeated three times, and the Petri dishes’ data were taken daily for four consecutive days in a week. The experiment was repeated weekly, changing the incubation temperature. Radicles were measured daily. When seeds reached a length of one millimeter, they were considered to have germinated. At the end of the experiment, (96 h), the germinated seeds were pulled out and measured for several germination characteristics. The germination characteristics for the TT, HT, and HTT models were established following a repeated probit regression analysis [12,16,26,34]. The sub- and supra-optimal *T_s_* were obtained from the following equation based on the HTT concept.

### 2.2. Data Analysis

The germination data were evaluated using the concept of TT, HT, and HTT models [35,36], through a repeated probit regression analysis. The germination rate (GR) for each percentile at each *T* or *Ψ* was calculated as the inverse of the germination time.

### 2.3. Thermal Time (TT)

We quantified the germination time data of constant *T_s_* at each *Ψ* using the concept of TT.

(1) $$\theta T1=\mathrm{TTsub}=\left(T-T_{b}\right)t_{g}\;\mathrm{at}\;\mathrm{sub-optimal}\;T_{s}$$

(2) $$\theta T2=\mathrm{TTsupra}=\left(T_{c\left(g\right)}-T\right)t_{g}\;\mathrm{at}\;\mathrm{supra-optimal}\;T_{s}$$

Thus, the germination rate is inversely related to the time of seed emergence, and Equations (1) and (2) may be represented as Equation (3):

(3) $$\mathrm{GR}=1/t_{g}=\left(T-T_{b\left(g\right)}\right)/\;\theta T$$

where *θT*1, *θT*2, *T*, and *T_b_*_(*g*)_ stand for the thermal time constants (°C h), expected temperature for seed germination, base temperature for germination fraction, and the time to germination fraction, respectively.

### 2.4. Hydrotime (HT)

The HT model was used to analyze the SG response to *Ψs* and accelerated aging [29]. Likewise, as in the TT model, *θ*H calculates the relation between the germination rate and solute potential. The HT model may be exhibited as follows:

(4) $$\theta H_{\left(g\right)}=\left(\psi -\psi_{b}\right)t_{g}$$

(5) $${\mathrm{GR}}_{\left(g\right)}=1/t_{g}=\left(\psi -\psi_{b}\right)/\theta H$$

where *θ*H, *Ψ*, *Ψ_b_*_(*g*)_, *t*_g_, and GR_(*g*)_ stand for the hydrotime constant (MPa h), actual water potential, the base water potential of germination fraction g (%), the time of seed population for the emergence of radicle, and the actual time to germination fraction g, respectively.

### 2.5. Hydrothermal Time Model (HTT)

We can merge the TT and HT models into a hybrid HTT model for predicting and describing SG behaviors to different *Ψ* and *T*. According to the HTT model, the germination time course at all *T_s_* and *Ψ* (from *T_b_* to *T_o_*) can be calculated as follows:

(6) $$\theta \mathrm{HTT}=\left(\Psi –\Psi_{b\left(g\right)}\right)\;\left(T-T_{b}\right)t_{g}$$

(7) $$\theta \mathrm{HTT}=\left[\Psi -\Psi_{b\left(g\right)}-\left(kT\;\left(T-T_{o}\right)\right)\right]\;\left(T-T_{b}\right)t_{g}$$

where *θ*HTT, *T_o_*, and *kT* stand for the HTT constant (MPa h), the optimum temperature for germination fraction, and the Boltzmann constant, respectively.

### 2.6. Germination Parameters

Based on the germination rate, root length, shoot lengths, leaf lengths, and the fresh and dry weights of plants, the following germination indices have been calculated.

#### 2.6.1. Germination Energy (GE)

The GE of plants was calculated following standard procedure [37].

(8) $$\mathrm{GE}=\frac{X_{1}}{Y_{1}}+\left(\frac{X_{2}-X_{1}}{Y_{2}}\right)+\left(\frac{X_{n}-X_{n-1}}{Y_{n}}\right)$$

Here *X*_1_, *X*_2_ and *X_n_* represent the frequency of germinated seeds on the first day, second day, and so on. Additionally, *Y*_1_, *Y*_2_ and *Y_n_* are the number of days from sowing to the first, second, and up to the last day’s count.

#### 2.6.2. Mean Germination Time (MGT)

The MGT index, which measures the speed with which seeds are emerging within a population, was calculated according to the following equation [38]:

(9) $$\mathrm{MGT}=\frac{\in \mathrm{fx}}{\in f}$$

where f represents seeds that germinated on day X.

#### 2.6.3. Mean Germination Rate (MGR)

The following formula was used to determine the MGR [39]:

(10) $$\mathrm{MGR}=\frac{1}{Mean\;Germination\;Time}$$

#### 2.6.4. Coefficient of Variation of Germination Time (CV_t_)

The value of CV_t_ was calculated according to the following equation [40]:

(11) $${\mathrm{CV}}_{t}=\frac{St}{t}\times 100$$

where *St* and *t* stand for the standard deviation of the germination time and mean germination time, respectively.

#### 2.6.5. Coefficient of Velocity of Germination (CVG)

The CVG represents the rate at which seeds germinate, and it will enhance with an increase in the frequency of germinating seeds. All sown seeds should germinate within the first 24 h to achieve the highest theoretical CVG. The following formula was used to calculate the CVG values [37]:

(12) $$\mathrm{CVG}=\frac{N1+\;N2+\;N3\ldots \mathrm{Nx}}{100}\times N1T1\ldots \mathrm{NxTx}$$

In this example, N stands for the number of seeds that germinate each day, and T stands for the time between sowing and germination of the N number of seeds.

#### 2.6.6. Germination Index (GI)

The value of the GI indicates the rate and speed of seed germination and was calculated using the following equation [41]:

(13) $$\mathrm{GI}=\left(10\times n1\right)+\left(9n\times n2\right)\ldots (1n\times 10)$$

where n1, n2, …, n10 illustrate the frequency of seed germination on the first, second, and last days of the experimental trial.

#### 2.6.7. Germination Rate Index (GRI)

The value of the GRI that describes the percentage of germination on certain days and at certain times was calculated according to the following equation [42]:

(14) $$\mathrm{GRI}=\frac{G1}{1}+\frac{G2}{2}+\frac{G3}{3}\ldots \frac{\mathrm{Gx}}{x}$$

where G1 and G2 represent the PG on the first and second day after sowing, respectively, and Gx represents the final GP.

#### 2.6.8. Seed Vigor Index-1 (SVI-1)

From each pot, the length of three seedlings was measured (cm) and calculated according to the following method [43]:

(15) $$\mathrm{SVI}-1=\mathrm{Seedlings}\;\mathrm{length}\left(\mathrm{cm}\right)\times \mathrm{Seed}\;\mathrm{Germination}\;\%\;\mathrm{age}$$

#### 2.6.9. Seed Vigor Index-2 (SVI-2)

Dry weight was calculated for three seedlings from each pot using an electrical balance. The values were then put into an equation and multiplied by the GP, using the following equation [44]:

(16) $$\mathrm{SVI}-2=\mathrm{Seed}\;\mathrm{dry}\;\mathrm{weight}\;\left(\mathrm{mg}\right)\times \mathrm{Seed}\;\mathrm{Germination}$$

#### 2.6.10. Time to 50% Germination (T_50%_)

The value of T_50%_ that illustrates the time needed for 50% of a seed to germinate was quantified by the following equation [45]:

(17) $$T50_{\%}=\frac{\mathrm{ti}\;+\left(N/2-\mathrm{ni}\right)\left(\mathrm{tj}-\mathrm{ti}\right)}{\left(\mathrm{nj}-\mathrm{ni}\right)}$$

where N represents the number of emerged seeds, and nj and ni represent the number of seeds that emerged after adjacent counts between tj and ti, where ni* N/2 > Nj.

#### 2.6.11. Root-Shoot Ratio (RSR)

We calculated the RSR using the following equation after the roots and shoots were dried in the oven for 24 h [43]:

(18) $$\mathrm{RSR}=\frac{root\;dry\;weight}{shoot\;dry\;weight}$$

#### 2.6.12. Germination Percentage (GP)

The GP illustrates the number of seeds germinated per 100 seeds sown in each pot, calculated using the following equation [38]:

(19) $$\mathrm{GP}=\;\frac{Final\;number\;of\;seedlinsg\;emerged}{Total\;number\;of\;seeds\;sown}\times 100$$

### 2.7. Statistical Analysis

We conducted a linear regression built with IBM^®^ SPSS^®^ Statistics Software Version 26.0 and SigmaPlot 11.0 to examine the effects of *T* (thermal model), *ψ* (hydrotime model), and their combined effects, i.e., *T* × *ψ* (hydrothermal time model). The basic statistical calculations were performed using Microsoft Excel. The values of σ*Ψ_b_*; *Ψ_b_*_(50)_, *R^2^*, SE, F, *t*-test, and Sig were calculated following a linear probit regression analysis in SPSS. Moreover, OriginPro^®^ 2021 Software (OriginLab Corporation, Northampton, MA, USA) was used to plot graphs of germination fractions against rapid aging duration, and germination characteristics against *T*.

## 3. Results

**Figure 1:**
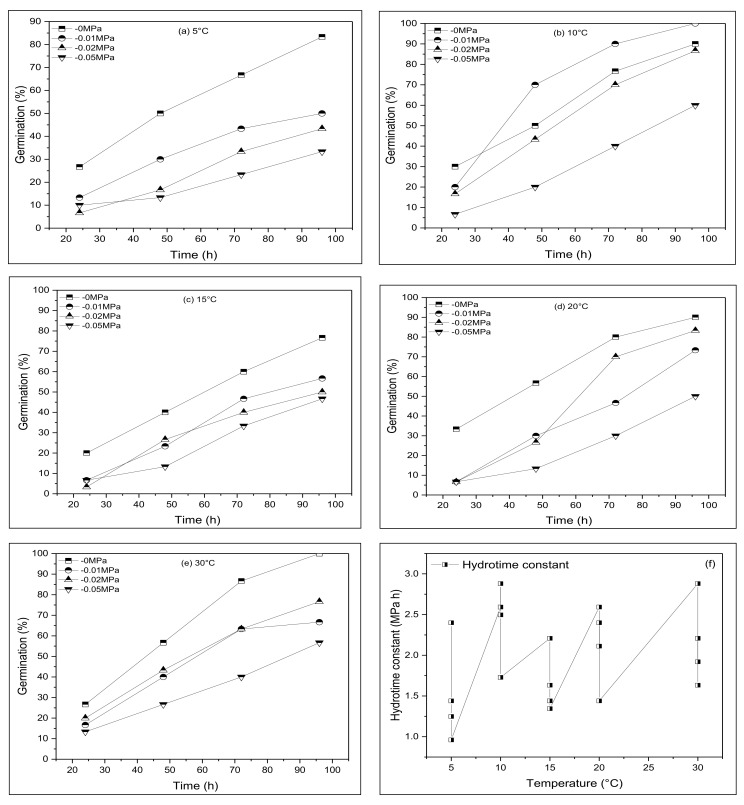
A cumulative germination fraction for *Eruca sativa* Mill. at (**a**) 5 °C, (**b**) 10 °C, (**c**) 15 °C, (**d**) 20 °C, (**e**) 30 °C, having different water potentials, (**f**) changes in halotime constant (*θ*H) as a function of temperature (*T*) for *Eruca sativa* Mill. symbols indicate water potential and lines indicate cumulative germination.

The final germination percentage (GP) of the rocket was greatly influenced by the fluctuating water potential (*ψ*) and temperature (*T*), as well their interactions (*ψ* × *T)* (*p* ≤ 0.05). The germination rate (GR) increased by 95% upon the rising temperature within the range of 20–30 °C. In contrast, the GR was recorded at 5 and 20 °C (~10 °C). In general, the GR appeared more responsive to changes in *ψ* than *T*. The highest GP was displayed at 30 °C in (0 MPa) and the minimum was at 5 and 20 °C. At each *ψ* and *T*, the germination percentage decreased as *ψ* decreased (Figure 1a–e). Throughout the experiment, the highest daily GP was reported on the last day under the distilling water (0 MPa). At *ψ* equal to (−0.02 MPa), zero growth was shown at the *T_s_* of 5, 10, 15, and 30 °C and seed germination (SG) was <20% at 5, 15, and 20 °C. The results confirmed that the value of *θT*1 was substantially high at 30 °C in (0 MPa) and at a minimum at 5 °C in *ψ* (−0.05 MPa) (Table 1). The value of θ*T*2 was at a maximum at 10 °C (−0.02 MPa). The maximum values of *θ*H and *θ*HTT were noticed at 10 and 30 °C, respectively. Moreover, the HTT model illustrated that the impact of *ψ* on seed germination was moderately high in comparison to *T*. Water potentials and seed population germination fractions were plotted against a constant *T* in the data set of *θ*H (Figure 1f). When the GR was exhibited against contrasting *T* percentiles, a linear rise in the GR_(*g*)_ pattern was observed at an optimum temperature (*T_b_*) and a decrease below the base temperature (*T_o_*). The GR_(*g*)_ of the HT model exhibited an increase at 5 °C in (−0.05 MPa).

The TT model well represented the kinetic of germination for the *ψ* range (0 to −0.05 MPa) and rising *T* at sub-optimal and supra-optimal. Moreover, the TT model was clinging to germination fraction data under distilled water with an increasing R^2^ value (0.829). Furthermore, GR_(*g*)_ responses were applied to calculate the ceiling, optimum, and base temperatures (Table 2). The consistency in σ*ψ_b_* showed that this change was maintained across all *T* and *ψ* level combinations, and similarly the least base water potential at 50% germination value (−0.18) was observed at 30 °C. The F value also showed an asymmetrical etiquette pattern, having no linear pattern, except that which was calculated to be high at 20 °C (Table 2). Our study showed that the minimum temperature observed (*T_b_*) for *E. sativa* was 5°C; below this threshold, the germination rate dropped and the plant’s physiological processes became harder to sustain. Optimal temperatures (*T_o_*) and ceiling temperatures (*T_c_*) were determined to be 30 °C (Table 3).

**Figure 2:**
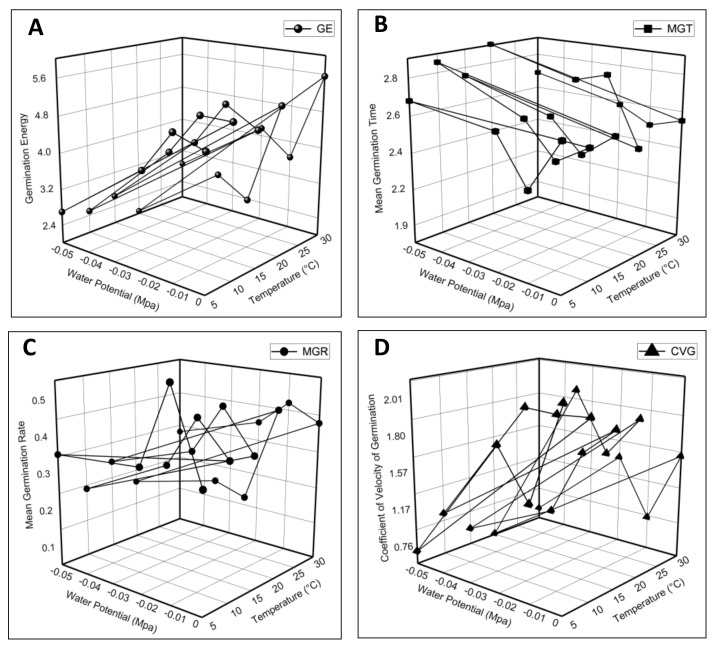
The effect of the water potential and temperature on (**A**) germination energy; (**B**) mean germination time; (**C**) mean germination rate; and (**D**) coefficient of the velocity of germination of *Eruca sativa* Mill.

**Figure 3:**
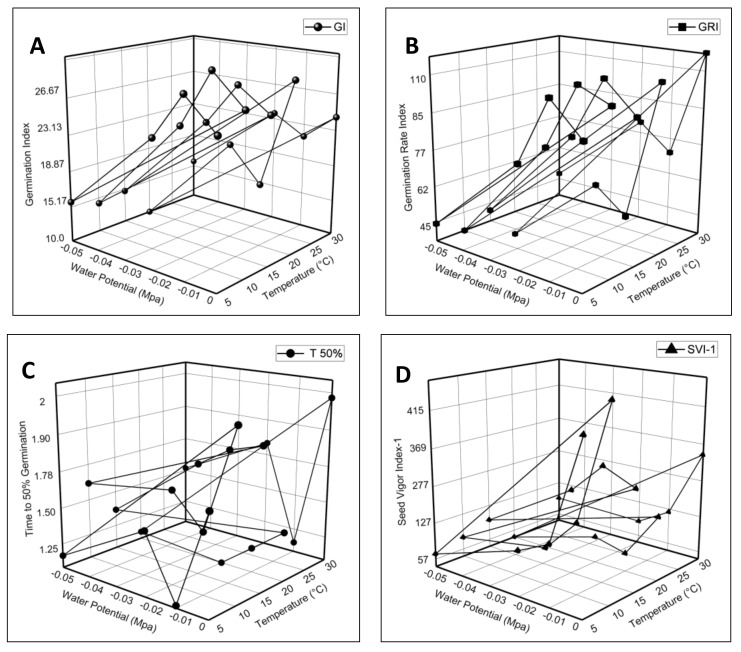
The effect of water potential and temperature on (**A**) germination index; (**B**) germination rate index; (**C**) time to 50% germination; and (**D**) seed vigor index-I of germination of *Eruca sativa* Mill.

The current HTT model experiment demonstrated that fluctuating *ψ* and *T* had a significant impact on *E. sativa* germination characteristics. The data of the germination parameters plotted against *ψ* and *T* were presented in 3D graphs. The results of the Petri dish experiment showed that the highest GE and GP were observed in seeds subjected to 30 °C (0 MPa), and a minimum at 20 °C in (−0.05 MPa) (Figure 2A). A maximum MGT was observed at 20 °C in (−0.01 MPa), and the minimum value was observed at 5 °C in −0.01 MPa (Figure 2B). Furthermore, the highest MGR reported in seeds was subjected to 5 °C in (−0.01 MPa), and the lowest at 10 °C in (−0.05 MPa) as shown in Figure 2C. The value of the CVG was at a maximum at 5 °C in distilled water, and a minimum at 20 °C in (−0.05 MPa) (Figure 2D). Moreover, the results also revealed the maximum GI value reported at 10 °C in (−0.01 MPa), and the lowest at 20 °C in (−0.05 MPa) as shown in Figure 3A.

**Figure 4:**
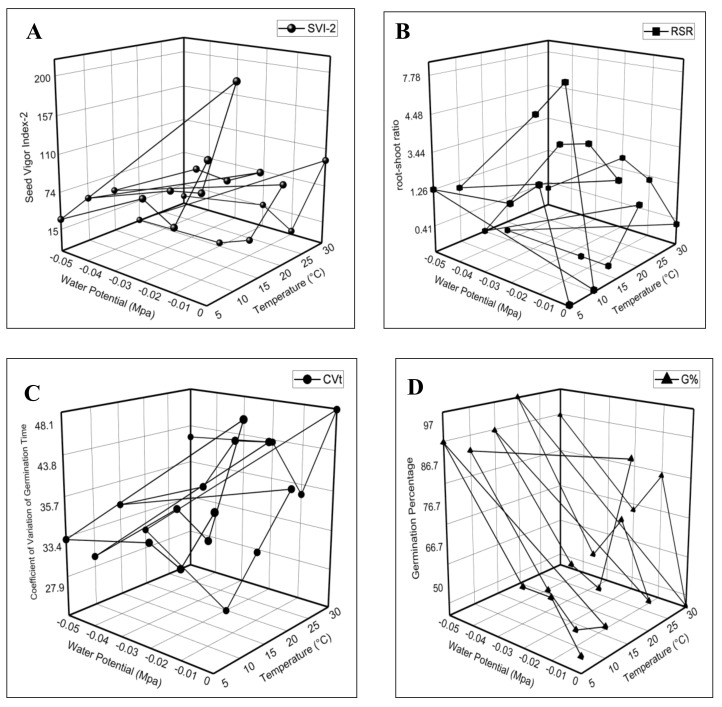
The effect of water potential and temperature on (**A**) seed vigor index-2; (**B**) root-shoot ratio; (**C**) coefficient of variation of germination time; and (**D**) germination percentage of germination of *Eruca sativa* Mill.

The maximum value of the germination rate index (GRI) was in seeds at 30 °C in (0 MPa) and the minimum value was at 10 °C in (−0.05 MPa) (Figure 3B). The results revealed the higher T_50%_ and coefficient of variation of the germination time (CV_t_) value in seeds treated with 30 °C in (0 MPa), and a minimum recorded at 20 °C in (−0.02 MPa) (Figure 3C and Figure 4C). Seed vigor indexes 1 and 2 recorded the maximum value at 10 °C in (0 MPa) and the minimum at 20 °C in (−0.05 MPa) (Figure 3D and Figure 4A). In addition, the highest root-shoot ratio (RSR) recorded was in seeds subjected to 10 °C in (−0.01 MPa), and the lowest was at 15 °C in (−0.05 MPa) (Figure 4B). Our findings illustrate that the germination characteristics were decreased when the water potential declined at each temperature, and these were at a maximum at the optimum temperature.

## 4. Discussion

Identifying the optimal geographical area, where a species can germinate and thrive, requires examining germination patterns under various environmental conditions. In this respect, mathematical models (TT, HT, and HTT models) are effective in quantifying the influence of these stress factors on seed germination [30]. Temperature (*T*) is one of the key environmental factors that impact seed germination in numerous plant species [46,47,48]. In addition, water stress is another abiotic stress factor that restricts early seedling and seed germination [49].

Several studies developed hydrotime (HT), thermal time (TT), and hydrothermal time (HTT) models as effective methods to describe and predict seed population germination responses under different environmental factors [20,34,50,51,52]. These models are common tools for both agronomical and basic research because they are simple and provide a direct biological interpretation of the parameters.

In the present study, we evaluated the response of *E. sativa* seeds to fluctuating *Ψ* and *T* using a hydrothermal model, by exploiting its flexibility for providing physiological cues that may be utilized in the future management of this crop species. The results of our study showed that increasing temperatures within the range of 20–30 °C resulted in an increase of 95% in germination, and the lowest value displayed a 25% decrease at 5 and 20 °C (~10 °C). The maximum growth rate was found at 30 °C, and the minimum was found at 5 °C. These findings are consistent with several previous studies [23,26,34,48,53,54]. According to them, agronomic parameters are affected significantly by *T* and *Ψ*, and when the *Ψ* becomes more negative, both the rate and percentage of germination decrease (if they do not become completely inhibited) due to the water deficit [55,56]. An explanation for the decrease in GP could be the thermal denaturation of essential amino acids that play a significant role in seed germination [57]. The declining pattern of GP coincides with a reduction in *Ψ*, which can lead to a decrease in water potential and a corresponding reduction in water resources [58,59].

In our study, the minimum temperature (*T_b_*) observed for *E. sativa* was 5 °C. It is an essential cardinal temperature threshold below which the germination rate drops and the physiological processes of a plant are more difficult to sustain; thus, *T_b_* must be considered in the development of a crop simulation model and determination of the optimum growing period [60]. In general, seeds are incubated at a constant *T* under laboratory conditions; however, they may face the problem of *T* fluctuations at the seedlings’ emergence stage in the field conditions. Seeds exposed to a changing temperature accumulated fewer thermal hours than those exposed to a constant temperature [61]. During the present study, the optimum (*T_o_*) and ceiling (*T_c_*) temperatures were both recorded at 30 °C. There are approximately three cardinal temperatures, including the ceiling (*T_c_*) and base (*T_b_*) temperatures, at which germination rates are minimal; and the optimal temperature (*T_o_*) at which high germination occurs immediately [62].

Further, the *T_o_* (30 °C) observed in the current study is comparable to the *T_o_* ranges between 25 and 28 °C (30 °C) observed in previous studies for watermelons [63,64,65]. Further, the maximum hydrotime constant (*θ*H) value was recorded at 10 °C. The GR_(*g*)_ of the HT concept exhibited a considerable increase at 5 °C in (−0.05 MPa). Despite *θ*H and GR_(*g*)_ being in contradiction with observations reported for *Sesamum indicum* [34] and potatoes [66], the decrease in water potential was associated with an increase in GR_(*g*)_ values, which are in agreement with the previous results for *Melissa officinalis* [67] and *Citrullus lanatus* [30]. Researchers and breeders used the *θ*H values to grade cultivars based on their susceptibility to *ψ*. In addition, the consistency in σ*ψ_b_* showed that this alteration was sustained at all combinations of *T* and *ψ* values, and likewise, the lowest base water potential at 50% germination value (−0.18) was recorded at 30 °C. The *ψ_b_*_(*g*)_ is an indicator that represents uniform changes in seed germination within a seed lot [24]. The results of several studies suggest that *ψ_b_*_(*g*)_ values are minimal at *T_o_* and increase linearly (e.g., become more positive) at supra-optimal *T_s_*, as reported for the tomato [68], potato [66], carrot and onion [51], zucchini [69], and watermelon [30].

TT and HT concepts have been effectively applied to explain SG attributes at *T* (sub-and supra-optimal) and *ψ*. However, the TT concept demonstrated an error at sub-optimal *T_s_* by failing to predict the decrease in GR when *T_s_* > *T_o_*. Therefore, to overcome this problem, Bradford developed the HTT model [67]. Currently, the HTT concept can be used to examine how abiotic factors (*T* and *ψ*) interactively influence the SG in seed lots [69,70]. In the present study, the maximum *θ*HTT value was recorded at 30 °C. The HTT model investigated for *Polylepis besseri* had predicted that the values of *T_b_*, *T_o_*, and *T_c_* were 3.08, 21.78, and 27.9 °C, respectively [71]. Based on the HTT model, the comparison results indicated that seed germination is more affected by water potential than by temperature.

The results showed that germination parameters like GE, CV_t_, GP, GRI, and T_50%_ were observed to be high in seeds grown at 30 °C and having zero water potential. The germination characteristics decreased when the water potential declined at each temperature, and these were at a maximum at the optimum temperature. Low *Ψ* can inhibit the chemical reactions and physiological mechanisms in the seed [69,72]. There is a particular significance of water stress in crop management because these factors represent the component of greatest trouble for both farmers and customers [73,74]. Therefore, using cardinal temperatures, germination results, and the hydrothermal time model (HTT) could reveal the response of seed germination to fluctuating *ψ* and *T*, independently or interactively (*ψ* × *T*).

## 5. Conclusions

The maximum GP was observed at 30 °C (0 MPa), and the minimum at 5 and 20 °C. The cardinal *T_s_* followed the pattern of 30 °C for *T_c_*, 30 °C for *T_o_*, and 5 °C for *T_b_*. Moreover, the *θ*H and *θ*HTT values were measured at 10 and 30 °C, respectively. Using the TT and HT models, the maximum *θT*1 and *θT*2 values were recorded in (0 and −0.02 MPa) at 30 and 10 °C; *Ψ_b_*_(50)_ in (−0.18 MPa); and the σ*Ψ_b_* value in (−0.13 MPa) at k_T_ (0.104 MPa) °C h^−1^. The germination parameters like GE, CV_t_, GP, GRI, and T_50%_ were observed to be high in seeds grown at 30 °C having zero water potential. As a result of reducing the water potential at all temperatures, germination was either retarded or inhibited. Therefore, using cardinal temperatures, germination results, and the hydrothermal time model (HTT) could reveal the response of seed germination to fluctuating *ψ* and *T*, independently or interactively (*ψ* × *T*), in the face of future climatic changes. However, the parameters of the model should analyze the physiological response of rocket seed populations under the influence of different abiotic factors for predicting the future germination time courses.

## Figures and Tables

**Table 1:** The estimated parameters of the hydro and thermal time models to describe *Eruca sativa* Mill. seed germination under fluctuating different temperatures (*T_s_*) and water potentials (*ψ_s_*).

T (°C)	*ψ* (MPa)	TTsub (*θT*1)	TTsupra (*θT*2)	*θ*H (MPa h)	*θ*HTT (MPa h)	Hydro Time GR_(*g*)_	Thermal Time GR_(*g*)_
5 °C	0	1600	2000	2.4	48	0.013	0.01
	−0.01	960	1200	1.44	28.8	0.021	0.02
	−0.02	832	1040	1.248	24.96	0.024	0.02
	−0.05	640	800	0.96	19.2	0.031	0.03
10 °C	0	1728	2160	2.592	51.84	0.012	0.01
	−0.01	1920	2400	2.88	57.6	0.010	0.01
	−0.02	1664	2080	2.496	49.92	0.012	0.01
	−0.05	1152	1440	1.728	34.56	0.017	0.02
15 °C	0	1472	1840	2.208	44.16	0.014	0.01
	−0.01	1088	1360	1.632	32.64	0.018	0.02
	−0.02	960	1200	1.44	28.8	0.021	0.02
	−0.05	896	1120	1.344	26.88	0.022	0.02
20 °C	0	1728	2160	2.592	51.84	0.012	0.01
	−0.01	1408	1760	2.112	42.24	0.014	0.01
	−0.02	1600	2000	2.4	48	0.013	0.01
	−0.05	960	1200	1.44	28.8	0.021	0.02
30 °C	0	1920	2400	2.88	57.6	0.010	0.01
	−0.01	1280	1600	1.92	38.4	0.016	0.02
	−0.02	1472	1840	2.208	44.16	0.014	0.01
	−0.05	1088	1360	1.632	32.64	0.018	0.02

T (Temperatures); *ψ* (Water potential); TTsub (Thermal time constant at sub-optimal temperature); TTsupra (Thermal time constant at supra-optimal temperature); *θ*H (Hydrotime constant); *θ*HTT (Hydrothermal time constant); GR (Germination rate).

**Table 2:** Estimation of hydrotime model parameters for *Eruca sativa* using non-linear regression.

Temperature	*ψ*_b(50)_ (MPa)	σ*ψ_b_* (MPa)	*R^2^*	*R*	SE	F	Sig.
5 °C	−0.13	0.142	0.695	0.834	1.464	4.559	0.16
10 °C	−0.15	0.153	0.815	0.903	0.901	8.793	0.09
15 °C	−0.19	0.176	0.640	0.800	0.991	3.558	0.20
20 °C	−0.11	0.187	0.829	0.911	0.883	9.702	0.08
30 °C	−0.21	0.133	0.647	0.805	1.338	3.673	0.19

*ψ*_b(50)_ (Base water potential at 50 percentile); σ*ψ_b_* (Standard deviation in *Ψ_b_*); *R^2^* (Coefficient of determination); SE (Standard error); F (Variability between different means); Sig (Level of significance).

**Table 3:** Estimated germination and cardinal temperature values for *Eruca sativa* Mill. using the hydrothermal time model.

Variables	*Eruca sativa* Mill.
	Hydrothermal time model parameters
*Ѱ_b_* (50) (MPa)	−0.18
σ*ψ_b_* (MPa)	−0.13
*θ*HTT (MPa °C h^−1^)	43.2
kT (MPa °C h^−1^)	0.104
	Cardinal temperatures
*T_b_* (°C)	5
*T_o_* (°C)	30
*T_c_* (°C)	30
*R^2^*	0.829

kT (Boltzmann constant); *R*^2^ (Coefficient of determination).
